# Factors Associated with Initial Mode of Breast Cancer Detection among Black Women in the Women's Circle of Health Study

**DOI:** 10.1155/2019/3529651

**Published:** 2019-07-04

**Authors:** Holly A. Szukis, Bo Qin, Cathleen Y. Xing, Michelle Doose, Baichen Xu, Jennifer Tsui, Yong Lin, Kim M. Hirshfield, Christine B. Ambrosone, Kitaw Demissie, Chi-Chen Hong, Elisa V. Bandera, Adana A. M. Llanos

**Affiliations:** ^1^Department of Biostatistics and Epidemiology, Rutgers School of Public Health, 683 Hoes Lane West, Piscataway, NJ, USA; ^2^Cancer Prevention and Control, Rutgers Cancer Institute of New Jersey, 195 Little Albany Street, New Brunswick, NJ, USA; ^3^Department of Health Behavior, Society and Policy, Rutgers School of Public Health, 683 Hoes Lane West, Piscataway, NJ, USA; ^4^Biometrics Division, Rutgers Cancer Institute of New Jersey, 195 Little Albany Street, New Brunswick, NJ, USA; ^5^Division of Medical Oncology, Rutgers Robert Wood Johnson Medical School, New Brunswick, NJ, USA; ^6^Division of Cancer Prevention and Control, Roswell Park Comprehensive Cancer Center, Buffalo, NY, USA; ^7^Department of Epidemiology and Biostatistics, SUNY Downstate School of Public Health, Brooklyn, NY, USA

## Abstract

Mammogram-detected breast cancers have a better prognosis than those identified through clinical breast exam (CBE) or through self-detection, primarily because tumors detected by mammography are more likely to be smaller and do not involve regional nodes. In a sample of 1,322 Black women, aged 40-75 years, diagnosed with breast cancer between 2002 and 2016, we evaluated factors associated with CBE and self-detection versus screening mammogram as the initial mode of breast cancer detection, using multivariable logistic regression models. Compared with screening mammogram, history of routine screening mammogram (OR 0.20, 95% CI: 0.07, 0.54) and performance of breast self-examination (BSE) (OR 0.31, 95% CI: 0.13, 0.74) before diagnosis were associated with lower odds of CBE as the initial mode of detection, while performance of CBEs before diagnosis (OR 11.04, 95% CI: 2.24, 54.55) was positively associated. Lower body mass index (<25.0 kg/m^2^ vs. ≥35.0 kg/m^2^: OR 2.46, 95% CI: 1.52, 3.98), performance of BSEs before diagnosis (less than once per month: OR 4.08, 95% CI: 2.45, 6.78; at least monthly: OR 4.99, 95% CI: 3.13, 7.97), and larger tumor size (1.0-2.0 cm vs. <1.0 cm: OR 2.92, 95% CI: 1.84, 4.64; >2.0 cm vs. <1.0 cm: OR 6.41, 95% CI: 3.30, 12.46) were associated with increased odds of self-detection relative to screening mammogram. The odds of CBE and self-detection as initial modes of breast cancer detection among Black women are independently associated with breast care and breast cancer screening services before diagnosis and with larger tumors at diagnosis.

## 1. Introduction

Although breast cancer incidence rates have been historically lower among African American/Black (hereafter “Black”) women than White women [1–5], recent data showed that incidence rates among these groups converged as of 2012 [6]. However, rates of incidence at ages <45 years [7] and mortality among Black women continue to be higher than among Whites [3–10]. National data from 2010-2014 [11, 12] demonstrated that breast cancer mortality was at least 41% higher among Blacks than Whites.

There are currently conflicting breast cancer screening guidelines, published by the United States Preventative Services Task Force (USPSTF) [13] and the American Cancer Society (ACS) [14], for women at average risk. The USPSTF recommends biennial screening among women aged 50-74 who are at average risk of breast cancer and the decision to begin screening prior to age 50 should be an individual one [13]. In contrast, the ACS strongly recommends annual mammography screening for women aged 45-54 and biennial mammography screening starting at age 55 [14]. Similar to the USPSTF position on mammography screening beginning at age 40, the ACS also recommends that women should have the choice to begin annual screening at this age. In 2009, the USPSTF concluded that there was insufficient evidence regarding the potential benefits and harms of clinical breast exams (CBEs) among women age 40 and older and recommended against teaching breast self-examination (BSE). The most recent USPSTF breast cancer screening guidelines did not update this language [13]. The ACS does not recommend CBE for breast cancer screening among average-risk women of any age and also recommends against systematic instruction of BSE, but suggests that all women should know how their breasts normally look and feel so that they are able to report noticeable changes to their healthcare provider in a timely manner [14].

Women's knowledge and awareness of the importance of mammography screening, as well as of breast cancer signs and symptoms are crucial, especially given changing and sometimes conflicting screening recommendations and evidence showing that initial mode of detection may be an independent prognostic factor for breast cancer. Breast cancers that are initially self-detected are commonly found to share more aggressive phenotypic characteristics (e.g., higher grade, hormone receptor negative, larger tumor size, positive lymph node status, and triple-negative breast cancer [TNBC] subtype) compared to those initially clinically detected through screening mammography or during routine physical exams performed by a healthcare provider [15–24]. Mammogram-detected breast cancers generally have more favorable clinicopathologic characteristics (e.g., lower grade, hormone receptor positive status, smaller tumor size, negative lymph node status, and luminal A subtype) than those originally detected through other means (e.g., self-detection, detection by partner, etc.) [15–25]. Evidence indicates that particular sociodemographic factors also differ when comparing women with screen-detected vs. self-detected breast cancers, including age, race/ethnicity, and educational attainment [23, 26]. Furthermore, evidence suggests that patients with mammogram-detected breast cancers have improved survival rates compared to those with self-detected cancers, specifically among those diagnosed with luminal A breast cancers [15, 16, 19, 22].

As there is currently a dearth of research regarding the factors associated with initial mode of breast cancer detection across diverse populations, the aim of this study was to identify specific factors (with a focus on sociodemographic characteristics, breast care characteristics prior to breast cancer diagnosis, and breast tumor clinicopathologic features at diagnosis) that might be associated with self-reported mode of initial breast cancer detection among a cohort of Black women with breast cancer.

## 2. Materials and Methods

### 2.1. Study Sample

We conducted case-case analysis of incident, primary breast cancers diagnosed among Black women 40-75 years, who were enrolled in the Women's Circle of Health Study (WCHS). As previously described [23, 27–29], WCHS is a case-control study conducted in metropolitan New York City (NYC) and ten counties in New Jersey (NJ). Breast cancer cases with histologically confirmed ductal carcinoma in situ or invasive breast cancer, who self-identified as either Black/African American or White/European American, were aged 20-75 years, were able to complete an interview in English, and had no history of cancer except nonmelanoma skin cancer, were eligible to participate. Recruitment in NYC was conducted between January 2002 and December 2008. Case identification was performed through hospitals within the 5 boroughs with the largest referrals for Black women. Recruitment in NJ began in March 2006 and is ongoing. Breast cancer cases residing in ten NJ counties are identified through rapid case ascertainment by the NJ State Cancer Registry (NJSCR). As of the start of the present analysis, approximately 83% of the breast cancer cases identified by NJSCR were deemed eligible for study participation and of these approximately 51% agreed to be contacted by research staff. Of those contacted, approximately 83% consented to participate in the study and completed the study interview. In this analysis, we included 1,322 Black breast cancer cases diagnosed between 2002 and 2016, who were 40-75 years old, were recruited in NYC and NJ, and had completed the baseline interview, including questions ascertaining their initial mode of breast cancer detection. This study was approved by the Institutional Review Boards of all participating institutions and all study participants provided written informed consent prior to enrollment in the study.

### 2.2. Data Collection

Data collection for the WCHS was conducted through in-person, interviewer-administered questionnaires at approximately 6-9 months after breast cancer diagnosis, assessing data relevant to the time of diagnosis as well as the time period of approximately 12 months before breast cancer diagnosis. Relevant to the current analysis, the baseline study questionnaire ascertained information on sociodemographic characteristics as well as established and probable breast cancer risk factors, including: family and personal medical history, reproductive history and hormone therapy (HT) use, cancer screening characteristics, and lifestyle exposures (e.g., tobacco smoke exposure, alcohol consumption, and physical activity). Anthropometric measurements (height, weight, and waist and hip circumference measures) were also taken at the in-person, baseline interview using standardized protocols and instruments [29]. Breast tumor clinicopathologic data were collected through medical and pathology records obtained from hospitals where breast cancer care was received.

Breast care characteristics approximately 12 months before breast cancer diagnosis were ascertained in the baseline questionnaire with the following questions: (1) Has a doctor ever recommended that you have a routine mammogram before your breast cancer diagnosis (yes, no)? (2) Before your diagnosis, had you ever had a routine mammogram (yes, no)? (3) Before your diagnosis, at what age or what year did you have your last routine mammogram? (4) Before your diagnosis, did you examine your breasts for lumps (yes, no)? (5) Before your diagnosis, did your healthcare provider examine your breasts for lumps (yes, no)? (6) How was your breast cancer first found (routine self-exam, accidental self-discovery, accidental discovery by a partner, routine physical exam by a doctor, routine screening mammogram, other)? Participants who reported “other” were excluded from subsequent analysis (n=321). Interval between breast cancer diagnosis and most recent routine screening mammogram was calculated as the difference (in years) between diagnosis age and age at last routine mammogram or as the difference (in years) between diagnosis date and date at last routine mammogram. Initial mode of breast cancer detection was classified into three categories: (1) screening mammogram (detected initially by a routine screening mammogram); (2) CBE (detected initially through CBE performed by a healthcare provider or through routine physical exam performed by a healthcare provider); and (3) self-detected (initially detected/palpated through routine BSE, through accidental self-discovery, or through accidental discovery by partner).

### 2.3. Data Analysis

Descriptive statistics were used to describe the study sample, overall and by initial mode of breast cancer detection. Chi-square tests (for categorical variables) and ANOVA models (for continuous variables) were used to compare sociodemographic, reproductive characteristics, medical history, breast care, and tumor characteristics, overall and by mode of breast cancer detection. Separate logistic regression models for each variable, adjusting for age, were used to estimate odds ratios (ORs) and 95% confidence intervals (CIs) of the overall associations between covariates of interest and initial mode of breast cancer detection. All variables that were significant in the age-adjusted analyses were included in a backward elimination procedure to obtain a final multivariable model, which was also age-adjusted. Any variable with* P *>0.10 was eliminated during the model selection procedure. The primary analysis focused on the associations of interest among women age 40-75 years. Sensitivity analysis was performed to assess the same associations among the subset of women age 50-75 years. Additionally, age-stratified multivariable analysis was performed separately for women who were 40-49, 50-59, and 60-75 years old. All reported* P* values were two-sided, and* P*≤0.05 was considered statistically significant. All analyses were performed using SAS v9.4 (SAS Institute, Cary, NC).

## 3. Results

### 3.1. Sociodemographic Characteristics of Study Sample and Differences by Initial Mode of Breast Cancer Detection

Sociodemographic characteristics, overall and by initial mode of breast cancer detection, are shown in Table 1. Among the 1,643 Black WCHS participants enrolled, 1,322 women age 40-75 years, with initial mode of detection classified as screening mammogram, CBE, or self-detection were included in the final analytic sample. Among this sample, 725 (54.8%) reported that their breast cancer was initially detected through screening mammogram, 63 (4.8%) through CBE, and 534 (40.4%) through self-detection. In the overall sample, approximately one-quarter of study participants was diagnosed with breast cancer at age <50 years. Approximately 44% was married or living as married, 35% had a college education or above, and 36% reported an annual household income of ≥$70,000. Nearly 60% percent reported having private health insurance, approximately 10% and 15% reported being covered by Medicaid or Medicare, respectively, 10% was uninsured, and about 6% had some “other” type of health insurance (e.g., school-provided, national government-provided [foreign country], health center care, health savings account, and Veterans Affairs coverage).

Only age (*P* <0.001) and type of primary health insurance at diagnosis (*P* <0.001) significantly differed by initial mode of breast cancer detection. The mean age at diagnosis among women whose breast cancers were detected through screening mammogram was approximately 57 years, while that of women reporting CBE and self-detection were 51 years and 50 years, respectively. In terms of insurance status at diagnosis, while there were similarly large proportions of women with private insurance across all modes of detection, the proportion of women who reported being uninsured was larger among those with self-detected breast cancers (14%) relative to those with mammogram-detected (7%) and CBE-detected (8%) cancers.

### 3.2. Reproductive Characteristics and Medical History of the Study Sample and Differences by Initial Mode of Breast Cancer Detection

As shown in Table 2, 61% of the overall study sample was postmenopausal, almost half (47%) reported being 12-13 years old at menarche, most (83%) had children, among whom 63% was <25 years old at their first live birth and approximately 46% reported having a history of breastfeeding. Almost 30% of the sample had a history of oral contraceptive use and a small proportion reported a history of postmenopausal HT use (17.5%). Less than 20% reported having a family history of breast cancer, and about half of all participants was obese. The distribution of menopausal status, HT use, and BMI differed significantly by mode of detection (all* P*-values <0.001). The proportion of postmenopausal women was smaller among those who reported their mode of detection was CBE or self-detection, relative to those who reported mammography-detection (43% and 48%, respectively, vs. 72%). Similarly, the proportion of women with a history of HT use was smaller among the CBE-detected and self-detected groups, relative to mammography-detected (10% and 11%, respectively, vs. 23%). Conversely, the proportions of women with BMI <25.0 kg/m^2^in the CBE-detected and self-detected groups were larger than among those in the mammography-detected group (25% and 26%, respectively, vs. 15%).

### 3.3. Breast Care Characteristics Prior to Breast Cancer Diagnosis and Tumor Clinicopathologic Features of the Study Sample and Differences by Initial Mode of Breast Cancer Detection

As shown in Table 3, the distributions of all breast care characteristics and breast tumor clinicopathologic features significantly differed by mode of detection. In brief, women who reported CBE or self-detection as the initial mode of breast cancer detection had more unfavorable breast care characteristics before diagnosis relative to those who reported that their breast cancer was detected through screening mammography. This included lower rates of receiving a doctor's recommendation for mammography, lower rates of ever having a mammogram, and longer time intervals between breast cancer diagnosis and most recent routine screening mammogram (P<0.0001). It should be noted that 18% of the study sample were outside the recommended screening interval, having an interval between diagnosis and the most recent routine screening mammogram that was longer than 2 years. Among women with CBE-detected and self-detected breast cancers, the proportions of higher grade, regional/distant SEER stage, larger size, positive lymph node status, presence of lymphovascular invasion, ER-negative status, and triple-negative subtype were larger relative to those with mammography-detected breast cancers, with women with self-detected cancers having the highest proportions (all* P-*values <0.001).

### 3.4. Age-Adjusted Associations between Sociodemographic, Reproductive, Clinical, Breast Care, and Tumor Clinicopathologic Characteristics with Initial Mode of Breast Cancer Detection

In the age-adjusted models (Table 4), premenopausal status (vs. postmenopausal status) was associated with increased odds of CBE-detection and younger age at menarche (<12 vs. >13 years) was associated with decreased odds of CBE-detection relative to detection by mammogram. Lower income (<$70,000 vs. ≥$70,000), being uninsured (vs. privately insured), and having a BMI <25.0 kg/m^2^ (vs. BMI ≥35.0 kg/m^2^) were associated with increased odds of self-detection compared to detection by mammogram. Having a history of HT use (vs. never use: OR 0.65, 95% CI: 0.46, 0.92) was associated with lower odds of self-detection compared to detection through screening mammogram.

As shown in Table 5, ever receiving a routine screening mammogram prior to breast cancer diagnosis (vs. never: OR 0.24, 95% CI: 0.11, 0.55) and performance of BSEs before diagnosis (at least monthly vs. no BSE performance) were associated with lower odds of having CBE as the initial mode of breast cancer detection (OR 0.37, 95% CI: 0.18, 0.78) relative to screening mammogram. Performance of CBEs before diagnosis, particularly having performed the last CBE more than a year before diagnosis (vs. no history of CBE) was associated with higher odds of CBE-detection (OR 4.23, 95% CI: 1.22, 14.62) relative to mammogram-detected breast cancer.

Later stage (regional/distant vs. localized), larger size (1.0-2.0 cm, >2.0 cm vs. <1.0 cm), and positive lymph node status (vs. negative status) were all associated with increased odds of CBE as the initial mode of breast cancer detection, relative to screening mammogram. Similarly, higher tumor grade (poorly differentiated vs. well/moderately differentiated), later stage, larger size, positive lymph node status, presence of lymphovascular invasion (vs. absence), ER- status (vs. ER+), and more aggressive breast cancer subtypes (namely ER-/PR-/HER2+ and ER-/PR-/HER2- vs. ER+/PR+/HER-) were associated with increased odds of breast cancer self-detection compared to detection through mammography.

### 3.5. Multivariable Regression Analysis of Factors Associated with Mode of Breast Cancer Detection

Results from the multivariable models were relatively consistent with those of the age-adjusted models (Table 6). After adjusting for all covariates, having a routine screening mammogram before breast cancer diagnosis (vs. never having a mammogram) was associated with 80% lower odds of CBE-detected breast cancer (OR 0.20, 95% CI: 0.07, 0.54) relative to mammogram-detected breast cancer. Performance of CBEs more than one year prior to breast cancer diagnosis (vs. no past performance of CBEs) was associated with more than 10-fold the odds of CBE-detected breast cancer (OR 11.04, 95% CI: 2.24, 54.55), while performing BSEs at least once per month (vs. no history of BSEs) was associated with almost 70% lower odds of CBE-detected breast cancer relative to mammogram-detected breast cancer (OR 0.20, 95% CI: 0.07, 0.54).

Women with a normal BMI had increased odds of self-detection relative to detection by mammogram (<25.0 kg/m^2^ vs. ≥35.0 kg/m^2^: OR 2.46, 95% CI: 1.52, 3.98). In contrast to the relationship with CBE-detection, having a history of performing BSEs (vs. never performing BSEs) was associated with 4-fold (BSEs performed less than once per month: OR 4.08, 95% CI: 2.45, 6.78) to 5-fold (BSEs performed at least once per month: OR 4.99, 95% CI: 3.13, 7.97) the odds of self-detection compared to detection by mammogram. Larger tumors were also associated with increased odds of self-detection (1.0-2.0 vs. <1.0 cm: OR 2.87, 1.81, 4.54; >2.0 vs. <1.0 cm: OR 6.87, 95% CI: 3.54, 13.34) compared to mammogram-detected.

In the sensitivity analysis we found that among women age 50-75, regional/distant SEER stage was associated with more than a 14-fold risk of CBE-detection relative to detection through mammography and history of mammography receipt was associated with lower odds of self-detection relative to mammography (Supplementary Table 1). These associations were not found in the primary analysis that included women age 40-75. In the age-stratified models (Supplementary Tables 2–4), we found that among women age 40-49, BMI <25.0 kg/m^2^ and 25.0-29.99 kg/m^2^ were associated with increased odds of self-detection relative to detection through screening mammogram (vs. BMI ≥35.0 kg/m^2^: OR 8.02, 95% C: 2.77, 23.21 and OR 3.12, 95% CI: 1.24, 7.88, respectively). Having a history of performing BSEs at least once per month before breast cancer diagnosis was associated with almost 5-fold increased odds of self-detection (OR 4.88, 95% CI: 2.00, 11.89); tumor size >2.0 cm (OR 10.57, 95% CI: 2.12, 52.71) and positive lymph node status (OR 5.41, 95% CI: 1.45, 20.15) were associated with increased odds of self-detection compared to detection through screening mammogram. These findings remained relatively consistent among women age 50-59 years, while among women in the 60-75 years age group, only performance of BSEs before breast cancer diagnosis and larger tumor size were found to be associated with increased odds of self-detection relative to detection by screening mammogram.

## 4. Discussion

Among Black women age 40-75 years, the odds of reporting CBE as the initial mode of breast cancer detection were independently associated with routine breast care characteristics prior to diagnosis (past history of routine screening mammography, performance of a CBE in the year before diagnosis, and having a history of performing BSEs monthly in the year before diagnosis) relative to the odds of reporting screening mammogram as the initial mode of detection. The odds of reporting self-detection as the initial mode of breast cancer were independently associated with lower BMI, history of HT use, having a history of CBEs and BSEs before diagnosis, and larger tumor size, relative to the odds of reporting screening mammogram as the initial mode of detection. Although age was an important confounder of the associations observed, the strong magnitudes of associations observed for breast care characteristics prior to diagnosis and larger tumor size with mode of breast cancer detection remained largely consistent, irrespective of age group (40-49, 50-59, or 60-75 years). The finding that larger tumors were associated with increased odds of CBE-detection and self-detection was not surprising and simply demonstrates that larger tumors are more easily palpable than smaller ones, and therefore can more easily come to the attention of a provider (upon performing a CBE) or the patient (through routinely performing BSEs). The finding that BMI <25 kg/m^2^ was associated with increased odds of self-detection supports prior literature showing that overweight/obese women might be less health conscious (i.e., less likely to participate in cancer prevention or related activities including as BSE, CBE, and/or mammography) [30–32]. Evidence also suggests that, compared to overweight/obese women, lower amounts of adipose breast tissue among those with BMI <25 kg/m^2^ might result in lower breast volume making it easier to detect breast abnormalities through palpation [33, 34]. A notable finding was that while having a routine screening mammogram before breast cancer diagnosis was significantly associated with lower odds of CBE-detected breast cancer, this factor was not significantly associated with odds of self-detection relative to the odds of mammography-detection.

Among the publications that have examined the characteristics associated with mode of breast cancer detection to date [15–25], most have not examined breast care characteristics occurring prior to breast cancer diagnosis as important predictors. The current study showed that among Black women 40-75 years old, for those that reported having a CBE within one year of diagnosis, there were almost 60% lower odds of breast cancer self-detection and among those who reported having a CBE more than one year before diagnosis, the odds of CBE as the initial mode of detection were 11-fold higher relative to screening mammogram. This is important because factors that affect whether Black women regularly experience opportunities for routine breast care services (i.e., receipt of frequent, routine CBEs) may be associated with socioeconomic status (e.g., income and health insurance status) and healthcare utilization factors (e.g., having a primary care provider, regularly having routine physical exams, etc.), as well as other factors that are not fully understood. Women's knowledge and awareness of the importance of breast health, as well as of breast cancer signs and symptoms are crucial, especially given conflicting screening recommendations and evidence showing that mode of detection may be an independent prognostic factor for breast cancer [15, 17–19].

In terms of self-detection, we found that past performance of BSEs was associated with 4-fold to 5-fold higher odds of self-detected breast cancer relative to mammogram-detected. This finding is particularly concerning in light of the lack of clarity regarding the benefits of BSEs and CBEs as suggested by current USPSTF guidelines, although these guidelines do not consider populations at increased risk for advance disease. Future research should explore the possibility that CBE and BSE can be used as an adjunct to routine mammography for earlier detection. Emerging data, from developing countries [35], suggest that BSE and CBE as adjuncts to screening mammography have effectiveness in clinical down staging among African women who present with later stage disease, similar to patterns observed among Black women in the US. The identification of additional integrated strategies to promote earlier detection among groups at increased risk for breast cancer mortality in the US might also include interventions at the health system level (e.g., improving patient-provider communication around the recommendation for screening mammogram, increasing physician's recommendations for screening mammograms across the board, etc.). In the sample of Black women 40-75 years old included in the current study, of the 725 women who reported that their breast cancer was initially detected through screening mammography, 85.5% reported ever receiving a doctor's recommendation for a mammogram and 86.8% reported ever having a screening mammogram before diagnosis. The proportion of women age 40-49 years who reported receiving a doctor's recommendation for a mammogram was lower (78.9%), although 89.1% of women in this age group reported having a routine screening mammogram before diagnosis. Receiving a doctor's recommendation for a mammogram before diagnosis was not significantly associated with mode of initial breast cancer detection in this study, but receipt of a mammogram prior to diagnosis was associated with reduced odds of initial detection through CBE and through self-detection compared to screening mammography. Evidence suggests that physicians' differential recommendation of screening mammography might contribute to differences in mammography receipt [36–38], thereby potentially contributing to variation in initial mode of breast cancer detection as observed herein.

Our consistent findings of breast care characteristics prior to diagnosis and tumor clinicopathologic features with initial mode of breast cancer detection among Black women in the primary analysis, in the sensitivity analysis, and in the age-stratified analysis suggest that these factors are important, irrespective of age at diagnosis. Since Black women are more likely than any other racial/ethnic group to be diagnosed at a younger age with tumors that are of later stage (and typically exhibit other features that denote more aggressive phenotypes) and have increased odds of breast cancer mortality [6, 8, 12, 39, 40], it is imperative that healthcare providers consult with their Black patients on what age to begin breast cancer screening and whether to receive screening mammograms annually or biennially. This is essential if a patient is considered to be at higher than average risk (i.e., having a strong family history of breast cancer or personal history of breast cancer, benign breast disease, or inherited* BRCA1* and/or* BRCA2* mutations). Postponing mammography screening could increase the possibility of self-detection and may result in detection of breast cancers that are more phenotypically aggressive, leading to poorer breast cancer survival rates [16, 17, 22]. Consequently, if routine and timely breast cancer screening is not equitably provided to all women, disparities in breast cancer outcomes will inevitably continue to widen.

This study has some limitations that should be considered. Presence of missing data for some of the breast cancer clinicopathologic characteristics (i.e., tumor grade, lymphovascular invasion presence, and molecular subtype) could have had an impact on our observed risk estimates. Another limitation is the potential for error and misclassification in some self-reported variables (e.g., sociodemographics, menopausal status, age at menarche, parity, age at first birth, history of breastfeeding, history of oral contraceptive use, history of HT use, and family history of breast cancer). In relation to self-reported mode of initial breast cancer diagnosis, we cannot rule out the possibility that women who reported that they routinely perform BSEs prior to their breast cancer diagnosis and/or reported that their breast cancer was first found through BSE could also be regular participants of mammography screening. There is also a strong possibility that patients who self-reported performing monthly BSEs were not given systematic instruction on how to perform BSEs and they may have responded that they regularly performed these exams, which might be what they considered a socially acceptable response. However, it is unlikely that recall errors or response bias contributed to variation in initial mode of detection, given that we expect women would know how their breast cancers were detected initially and report it accurately. Therefore, the resulting potential misclassification of initial mode of detection is likely to be random, which would actually lead to an underestimation of the true associations. It would be ideal to investigate the possibility of interval cancers being self-detected or CBE-detected between routine screening mammograms, which can be the focus of future analysis involving medical and pathology data for confirmation. Additionally, it is also essential to further understand whether the observed associations are due to inability of detecting some breast cancers earlier due to factors of a more biologic nature, which might lead to larger breast tumors at diagnosis. Conversely, more phenotypically aggressive breast cancers could grow faster and could be more prone to being found through CBE or BSE/self-detection, before a woman accesses mammography screening or shortly after having a mammogram with normal results. For example, triple-negative and HER2-positive tumors are known to occur as interval tumors (those detected within the period between a normal mammography screening and when the next mammogram is due) [41–43]. Despite these limitations, several statistically significant findings were observed.

Some of the strengths of this study include the utilization of a cohort of well-defined, Black breast cancer cases nested within a case-control study with detailed data available through collection and abstraction of medical and pathology records as well as through interviewer-administered questionnaires. An additional strength is that this study is one of the first to analyze predictors of mode of breast cancer detection among a population of Black women with varying socioeconomic/insurance status. By understanding how specific factors are associated with mode of breast cancer detection among minority women who have a demonstrated disadvantage in breast cancer survival outcomes, we can begin to better understand and develop integrated strategies for addressing disparities in breast cancer outcomes.

## 5. Conclusion

The findings from this study contribute to the limited literature on factors associated with mode of breast cancer detection, specifically among Black women. Our study found that higher odds of breast cancers being CBE-detected or BSE/self-detected among Black women 40-75 years old were associated with breast care characteristics before breast cancer diagnosis and larger tumor size. These findings highlight the need to improve the rates of early breast cancer detection to potentially reduce breast cancer mortality among populations that currently suffer disproportionate mortality, such as Black women. Healthcare providers must continue educating women on the importance of early breast cancer detection, as well as developing and implementing novel, integrated strategies that promote earlier detection, particularly among women who are at increased risk of being diagnosed with breast cancers having more aggressive phenotypes and who have increased risk of breast cancer mortality.

## Figures and Tables

**Table 1:** Sociodemographic characteristics of Black women age 40-75 years in the Women's Circle of Health Study (WCHS), overall and by initial mode of breast cancer detection.

	Overall	Screening mammogram	Clinical breast exam	Self-detection	*P*-value
	(N = 1322)	(n = 725)	(n = 63)	(n = 534)	
	*N (*%)	*n (*%)	*n (*%)	*n (*%)	
Age at diagnosis (years), mean±SD	54.19±10.7	57.35±9.2	51.44±11.1	50.17±11.1	*<0.001*
Age at diagnosis (years)					*<0.001*
40-49	325 (24.6)	147 (20.3)	17 (27.0)	161 (30.2)	
50-59	431 (32.6)	248 (34.2)	17 (27.0)	166 (31.1)	
60-75	449 (34.0)	318 (43.9)	17 (27.0)	114 (21.4)	
Marital status^a^					0.494
Married or living as married	576 (43.6)	326 (45.0)	25 (39.7)	225 (42.1)	
Unmarried	746 (56.4)	399 (55.0)	38 (60.3)	309 (57.9)	
Education					0.390
High school/vocational/technical graduate or below	538 (40.7)	311 (42.9)	25 (39.7)	202 (37.8)	
Some post-secondary education	327 (24.7)	167 (23.0)	13 (20.6)	147 (27.5)	
College graduate or above	456 (34.5)	246 (33.9)	25 (39.7)	185 (34.6)	
Annual household income^b^					0.224
<$70,000	765 (57.9)	400 (55.2)	37 (58.7)	328 (61.4)	
≥$70,000	480 (36.3)	282 (38.9)	21 (33.3)	177 (33.2)	
Primary health insurance at diagnosis^c^					*<0.001*
Private	775 (58.6)	413 (57.0)	39 (61.9)	323 (60.5)	
Medicaid	130 (9.8)	65 (9.0)	8 (12.7)	57 (10.7)	
Medicare	193 (14.6)	140 (19.3)	10 (15.9)	43 (8.1)	
Uninsured	131 (9.9)	51 (7.0)	5 (7.9)	75 (14.0)	
Other	81 (6.1)	50 (6.9)	1 (1.6)	30 (5.6)	
Missing	12 (0.9)	6 (0.8)	0 (0.0)	6 (1.1)	

^a^The unmarried category is composed of those who reported being single/never married, separated, divorced, or widowed.

^b^$70,000 is the median income among households in New Jersey and was used as the cut-point to dichotomize the income variable.

^c^The “other” insurance category includes forms of insurance reported by WCHS participants that did not fall into one of the four main categories above and included the following types of insurance coverage: school-provided, national government-provided [foreign country], spousal insurance, health center care, charity care, health savings account, and Veterans Affairs coverage.

**Table 2:** Reproductive characteristics and medical history of Black women aged 40-75 years in the WCHS, overall and by initial mode of breast cancer detection.

	Overall	Screening mammogram	Clinical breast exam	Self-detection	*P*-value
	(N = 1322)	(n = 725)	(n = 63)	(n = 534)	
	*N (*%)	*n(*%)	*n(*%)	*n(*%)	
Menopausal status					*<0.0001*
Premenopausal	516 (39.0)	203 (28.0)	36 (57.1)	277 (51.9)	
Postmenopausal	806 (61.0)	522 (72.0)	27 (42.9)	257 (48.1)	
Age at menarche (years)					0.324
<12	381 (28.8)	212 (29.2)	11 (17.5)	158 (29.6)	
12-13	618 (46.8)	338 (46.6)	32 (50.8)	248 (46.4)	
>13	323 (24.4)	175 (24.1)	20 (31.8)	128 (24.0)	
Parity					0.428
Nulliparous	231 (17.5)	117 (16.1)	9 (14.3)	105 (19.7)	
1-2 children	679 (51.4)	377 (52.0)	31 (49.2)	271 (50.8)	
≥3 children	412 (31.2)	231 (31.9)	23 (36.5)	158 (29.6)	
Age at first birth (years)^a^					0.605
<25	687 (63.0)	382 (62.8)	30 (55.6)	275 (64.1)	
25-30	242 (22.2)	130 (21.4)	15 (27.8)	97 (22.6)	
>30	162 (14.9)	96 (15.8)	9 (16.7)	57 (13.3)	
History of breastfeeding^a^					0.458
No	592 (54.5)	336 (55.5)	32 (59.3)	224 (52.3)	
Yes	495 (45.5)	269 (44.5)	22 (40.7)	204 (47.7)	
History of oral contraceptive use					0.685
No	933 (70.6)	505 (69.7)	42 (66.7)	386 (72.3)	
Yes	388 (29.4)	219 (30.2)	21 (33.3)	148 (27.7)	
History of hormone therapy use					*<0.0001*
No	1084 (82.0)	556 (76.7)	57 (90.5)	471 (88.2)	
Yes	231 (17.5)	166 (22.9)	6 (9.5)	59 (11.1)	
Family history of breast cancer					0.733
No	1075 (81.3)	584 (80.6)	52 (82.5)	439 (82.2)	
Yes	247 (18.7)	141 (19.5)	11 (17.5)	95 (17.8)	
Body mass index (kg/m^2^)					*<0.001*
<25.0	261 (19.7)	105 (14.5)	16 (25.4)	140 (26.2)	
25.0-29.99	389 (29.4)	226 (31.2)	17 (27.0)	146 (27.3)	
30.0-34.99	312 (23.6)	189 (26.1)	14 (22.2)	109 (20.4)	
≥35.0	359 (27.2)	204 (28.1)	16 (25.4)	139 (26.0)	

^a^Age at first birth and history of breastfeeding data are among parous women only.

**Table 3:** Breast care characteristics prior to breast cancer diagnosis and tumor clinicopathologic features among Black women aged 40-75 years in the WCHS, overall and by initial mode of breast cancer detection.

	Overall	Screening mammogram	Clinical breast exam	Self-detection	*P*-value
	(N = 1643)	(n = 725)	(n = 63)	(n = 534)	
	*N (*%)	*n(*%)	*n(*%)	*n(*%)	
*Breast care characteristics prior to breast cancer diagnosis*					
Ever received a doctor's recommendation for a mammogram before breast cancer diagnosis					*<0.001*
No	274 (20.7)	103 (14.2)	16 (25.4)	155 (29.0)	
Yes	1045 (79.1)	620 (85.5)	47 (74.6)	378 (70.8)	
Ever had a routine screening mammogram before breast cancer diagnosis					*<0.001*
No	174 (13.2)	37 (5.1)	16 (25.4)	121 (22.7)	
Yes	1148 (86.8)	688 (94.9)	47 (74.6)	413 (77.3)	
Interval between breast cancer diagnosis and most recent routine screening mammogram					*<0.001*
<1 year	112 (8.5)	66 (9.1)	4 (6.4)	42 (7.9)	
1 year	565 (42.7)	382 (52.7)	17 (27.0)	166 (31.1)	
>1 year	377 (28.5)	186 (25.7)	17 (27.0)	174 (32.6)	
Unknown	268 (20.3)	91 (12.6)	25 (39.7)	152 (28.5)	
Ever had a doctor perform a clinical breast exam(s) (CBE) before breast cancer diagnosis					*<0.001*
No	183 (13.8)	91 (12.6)	4 (6.4)	88 (16.5)	
Yes – last CBE performed within the last year	795 (60.1)	482 (66.5)	35 (55.6)	278 (52.1)	
Yes – last CBE performed more than one year ago	332 (25.1)	146 (20.1)	23 (36.5)	163 (30.5)	
Ever performed breast self-exams (BSEs) before breast cancer diagnosis					*<0.001*
No	283 (21.4)	197 (27.2)	25 (39.7)	61 (11.4)	
Yes – BSEs performed less than once per month	375 (28.4)	184 (25.4)	24 (38.1)	167 (31.3)	
Yes – BSEs performed at least once per month	657 (49.7)	338 (46.6)	14 (22.2)	305 (57.1)	
Ever diagnosed with a benign breast disease before breast cancer diagnosis					*0.042*
No	839 (63.5)	443 (61.1)	42 (66.7)	354 (66.3)	
Yes	476 (36.0)	281 (38.8)	20 (31.8)	175 (32.8)	
*Breast tumor clinicopathologic features*					
Tumor grade					*<0.001*
Well/moderately differentiated	608 (46.0)	357 (49.2)	36 (57.1)	215 (40.3)	
Poorly differentiated	483 (36.5)	178 (24.6)	22 (34.9)	283 (53.0)	
Unknown	231 (17.5)	190 (26.2)	5 (7.9)	36 (6.7)	
SEER summary stage					*<0.001*
In situ	166 (12.6)	144 (19.9)	2 (3.2)	20 (3.8)	
Localized	718 (54.3)	427 (58.9)	37 (58.7)	254 (47.6)	
Regional/distant	376 (28.4)	115 (15.9)	23 (36.5)	238 (44.6)	
Unstaged	15 (1.1)	5 (0.7)	1 (1.6)	9 (1.7)	
Missing	47 (3.6)	34 (4.7)	0 (0.0)	13 (2.4)	
Tumor size (cm)					*<0.001*
<1.0	509 (38.5)	413 (57.0)	15 (23.8)	81 (15.2)	
1.0-2.0	356 (26.9)	196 (27.0)	22 (34.9)	138 (25.8)	
>2.0	456 (34.5)	115 (15.9)	26 (41.3)	315 (59.0)	
Unknown	1 (0.1)	1 (0.1)	0 (0.0)	0 (0.0)	
Lymph node status					*<0.001*
Negative	814 (61.6)	500 (69.0)	38 (60.3)	276 (51.7)	
Positive	378 (28.6)	119 (16.4)	21 (33.3)	238 (44.6)	
Unknown	130 (9.8)	106 (14.6)	4 (6.4)	20 (3.8)	
Lymphovascular invasion present					*<0.001*
No	856 (64.8)	487 (67.2)	45 (71.4)	324 (60.7)	
Yes	207 (15.7)	59 (8.1)	11 (17.5)	137 (25.7)	
Unknown	259 (19.6)	179 (24.7)	7 (11.1)	73 (13.7)	
Estrogen receptor (ER) status					*<0.001*
ER+	972 (73.5)	573 (79.0)	56 (88.9)	343 (64.2)	
ER-	346 (26.2)	148 (20.4)	7 (11.1)	191 (35.8)	
Unknown	4 (0.3)	4 (0.6)	0 (0.0)	0 (0.0)	
Molecular subtype^a^					*<0.001*
ER+/PR+/HER2-	745 (56.4)	431 (59.5)	44 (69.8)	270 (50.6)	
ER+/PR+/HER2+	127 (9.6)	56 (7.7)	10 (15.9)	61 (11.4)	
ER-/PR-/HER2+	78 (5.9)	33 (4.6)	1 (1.6)	44 (8.2)	
ER-/PR-/HER2-	234 (17.7)	92 (12.7)	6 (9.5)	136 (25.5)	
Unknown	138 (10.4)	113 (15.6)	2 (3.2)	23 (4.3)	

^a^Molecular subtypes were classified using surrogate classifications, based on immunohistochemistry expression of ER and PR, and overexpression of HER2 (by immunohistochemistry and/or fluorescence in situ hybridization) as reported in pathology records.

**Table 4:** Age-adjusted associations of sociodemographic, reproductive characteristics and medical history with initial mode of breast cancer detection among Black women aged 40-75 years.

	Clinical breast exam compared to screening mammogram	Self-detection compared to screening mammogram
	Age-adjusted	Age-adjusted
	OR (95% CI)	OR (95% CI)
Marital status^a^		
Married or living as married	1.00 (Referent)	1.00 (Referent)
Unmarried	1.25 (0.70, 2.24)	1.27 (0.99, 1.63)
Education		
High school/vocational/technical graduate or below	0.97 (0.5, 1.86)	1.10 (0.82, 1.46)
Some post-secondary education	0.93 (0.43, 2.01)	1.31 (0.95, 1.81)
College graduate or above	1.00 (Referent)	1.00 (Referent)
Annual household income^b^		
<$70,000	1.37 (0.73, 2.58)	*1.46 (1.12, 1.91)*
≥$70,000	1.00 (Referent)	1.00 (Referent)
Primary health insurance at diagnosis^c^		
Private	1.00 (Referent)	1.00 (Referent)
Medicaid	1.30 (0.52, 3.26)	1.14 (0.75, 1.74)
Medicare	1.47 (0.62, 3.49)	0.78 (0.51, 1.18)
Uninsured	1.14 (0.38, 3.37)	*2.16 (1.44, 3.25)*
Other	0.27 (0.04, 2.04)	0.76 (0.45, 1.27)
Menopausal Status		
Premenopausal	*2.68 (1.07, 6.71)*	0.98 (0.66, 1.48)
Postmenopausal	1.00 (Referent)	1.00 (Referent)
Age at menarche (years)		
<12	*0.41 (0.17, 0.99)*	0.90 (0.65, 1.25)
12-13	0.87 (0.45, 1.66)	0.90 (0.67, 1.22)
>13	1.00 (Referent)	1.00 (Referent)
Parity		
Nulliparous	1.00 (Referent)	1.00 (Referent)
1-2 children	1.59 (0.59, 4.25)	0.88 (0.63, 1.24)
≥3 children	2.42 (0.88, 6.63)	1.10 (0.77, 1.59)
Age at first birth (years)		
Nulliparous	1.00 (Referent)	1.00 (Referent)
<25	1.84 (0.69, 4.91)	1.06 (0.75, 1.48)
25-30	2.20 (0.75, 6.46)	0.93 (0.62, 1.41)
>30	1.59 (0.49, 5.19)	0.69 (0.44, 1.09)
History of breastfeeding		
Nulliparous	1.00 (Referent)	1.00 (Referent)
No	2.29 (0.86, 6.08)	1.03 (0.73, 1.46)
Yes	1.54 (0.55, 4.28)	0.90 (0.63, 1.28)
History of oral contraceptive use		
No	1.00 (Referent)	1.00 (Referent)
Yes	0.62 (0.34, 1.12)	0.89 (0.68, 1.17)
History of hormone therapy use		
No	1.00 (Referent)	1.00 (Referent)
Yes	0.52 (0.21, 1.28)	*0.65 (0.46, 0.92)*
Family history of breast cancer		
No	1.00 (Referent)	1.00 (Referent)
Yes	1.05 (0.51, 2.16)	0.96 (0.7, 1.31)
Body mass index (kg/m^2^)		
<25.0	1.2 (0.52, 2.78)	*1.56 (1.09, 2.22)*
25.0-29.99	0.69 (0.31, 1.51)	0.79 (0.57, 1.09)
30.0-34.99	1.01 (0.48, 2.16)	0.72 (0.51, 1.02)
≥35.0	1.00 (Referent)	1.00 (Referent)

^a^The unmarried category is composed of those who reported being single/never married, separated, divorced, or widowed.

^b^$70,000 is the median income among households in New Jersey and was used as the cut-point to dichotomize the income variable.

^c^The “other” insurance category includes forms of insurance reported by WCHS participants that did not fall into one of the four main categories above and included the following types of insurance coverage: school-provided, national government-provided [foreign country], spousal insurance, health center care, charity care, health savings account, and Veterans Affairs coverage.

**Table 5:** Age-adjusted associations of breast care characteristics prior to breast cancer diagnosis and tumor clinicopathologic characteristics with initial mode of breast cancer detection among Black women aged 40-75 years.

	Clinical breast exam compared to screening mammogram	Self-detection compared to screening mammogram
	Age-adjusted	Age-adjusted
	OR (95% CI)	OR (95% CI)
*Breast care characteristics prior to breast cancer diagnosis*		
Ever received a doctor's recommendation for a mammogram		
No	1.00 (Referent)	1.00 (Referent)
Yes	0.70 (0.34, 1.47)	0.73 (0.53, 1.02)
Ever had a routine screening mammogram before breast cancer diagnosis		
No	1.00 (Referent)	1.00 (Referent)
Yes	*0.24 (0.11, 0.55)*	*0.38 (0.24, 0.60)*
Interval between breast cancer diagnosis and most recent routine screening mammogram		
<1 year	1.00 (Referent)	1.00 (Referent)
1 year	0.84 (0.24, 3.00)	0.71 (0.45, 1.12)
>1 year	1.99 (0.56, 7.03)	1.59 (1.00, 2.54)
Ever had a doctor perform a clinical breast exam(s) (CBE) before diagnosis		
No	1.00 (Referent)	1.00 (Referent)
Yes – last CBE performed within the last year	1.47 (0.43, 4.99)	*0.43 (0.30, 0.61)*
Yes – last CBE performed more than one year ago	*4.23 (1.22, 14.62)*	1.13 (0.76, 1.67)
Ever performed breast self-exams (BSEs) before breast cancer diagnosis		
No	1.00 (Referent)	1.00 (Referent)
Yes – BSEs performed less than once per month	1.04 (0.54, 2.02)	*3.06 (2.06, 4.54)*
Yes – BSEs performed at least once per month	*0.37 (0.18, 0.78)*	*3.42 (2.38, 4.93)*
Ever diagnosed with a benign breast disease before breast cancer diagnosis		
No	1.00 (Referent)	1.00 (Referent)
Yes	0.84 (0.46, 1.55)	0.90 (0.7, 1.17)
*Breast tumor clinicopathologic features*		
Tumor grade		
Well/moderately differentiated	1.00 (Referent)	1.00 (Referent)
Poorly differentiated	0.95 (0.50, 1.81)	*2.44 (1.86, 3.21)*
SEER summary stage		
In situ	*0.22 (0.05, 0.94)*	*0.22 (0.12, 0.37)*
Localized	1.00 (Referent)	1.00 (Referent)
Regional/distant	*2.93 (1.59, 5.39)*	*3.21 (2.28, 4.47)*
Tumor size (cm)		
<1.0	1.00 (Referent)	1.00 (Referent)
1.0-2.0	*3.54 (1.64, 7.65)*	*4.07 (2.85, 5.81)*
>2.0	*7.26 (3.41, 15.44)*	*14.64 (10.30, 20.80)*
Lymph node status		
Negative	1.00 (Referent)	1.00 (Referent)
Positive	*2.99 (1.62, 5.51)*	*3.43 (2.58, 4.56)*
Lymphovascular invasion present		
No	1.00 (Referent)	1.00 (Referent)
Yes	1.92 (0.88, 4.18)	*3.21 (2.24, 4.59)*
Estrogen receptor (ER) status^a^		
ER+	1.00 (Referent)	1.00 (Referent)
ER-	0.40 (0.16, 1.03)	*2.06 (1.57, 2.70)*
Molecular subtype^b^		
ER+/PR+/HER2-	1.00 (Referent)	1.00 (Referent)
ER+/PR+/HER2+	1.57 (0.69, 3.56)	1.50 (0.98, 2.31)
ER-/PR-/HER2+	0.32 (0.04, 2.43)	*1.87 (1.12, 3.11)*
ER-/PR-/HER2-	0.48 (0.17, 1.39)	*2.17 (1.56, 3.01)*

^a^As shown in Table 3, percent unknown for tumor characteristics in the overall study sample was as follows: tumor grade, 17.5%; SEER summary stage, 3.6%; tumor size (cm), 0.1%; lymph node status, 9.8%; lymphovascular invasion present, 19.6%; ER status, 0.3%; molecular subtype, 10.4%.

^b^Molecular subtypes were classified using surrogate classifications, based on immunohistochemical expression of ER and PR and overexpression or amplification of HER2 (by immunohistochemistry or fluorescence in situ hybridization) as reported in pathology records.

**Table 6:** Multivariable logistic regression analysis of the factors associated with mode of detection among Black women aged 40-75 years.

Characteristic	Clinical breast exam compared to screening mammogram	Self-detection compared to screening mammogram
	Multivariable-adjusted	Multivariable-adjusted
	OR (95% CI)	OR (95% CI)
Primary health insurance at diagnosis		
Private	1.00 (Referent)	1.00 (Referent)
Medicaid	0.99 (0.31, 3.15)	1.03 (0.59, 1.82)
Medicare	1.84 (0.63, 5.38)	0.83 (0.48, 1.43)
Uninsured	0.58 (0.14, 2.37)	1.38 (0.77, 2.47)
Other	0.31 (0.04, 2.63)	0.92 (0.46, 1.83)
Body mass index (kg/m^2^)		
<25.0	1.33 (0.49, 3.62)	*2.46 (1.52, 3.98)*
25.0-29.99	0.79 (0.32, 1.95)	1.07 (0.70, 1.64)
30.0-34.99	1.22 (0.50, 2.97)	0.80 (0.50, 1.26)
≥35.0	1.00 (Referent)	1.00 (Referent)
History of hormone therapy use		
No	1.00 (Referent)	1.00 (Referent)
Yes	0.61 (0.23, 1.63)	*0.62 (0.39, 0.97)*
Ever had a routine screening mammogram before breast cancer diagnosis		
No	1.00 (Referent)	1.00 (Referent)
Yes	*0.20 (0.07, 0.54)*	0.52 (0.27, 1.00)
Ever had a doctor perform a clinical breast exam(s) (CBE) before breast cancer diagnosis		
No	1.00 (Referent)	1.00 (Referent)
Yes – last CBE performed within the last year	4.33 (0.90, 20.83)	*0.48 (0.29, 0.79)*
Yes – last CBE performed more than one year ago	*11.04 (2.24, 54.55)*	1.28 (0.74, 2.20)
Ever performed breast self-exams (BSEs) before breast cancer diagnosis		
No	1.00 (Referent)	1.00 (Referent)
Yes – BSEs performed less than once per month	0.92 (0.42, 2.01)	*4.08 (2.45, 6.78)*
Yes – BSEs performed at least once per month	*0.31 (0.13, 0.74)*	*4.99 (3.13, 7.97)*
*Breast tumor clinicopathologic features*		
Tumor grade^a^		
Well/moderately differentiated	1.00 (Referent)	1.00 (Referent)
Poorly differentiated	0.65 (0.28, 1.47)	1.19 (0.80, 1.77)
SEER summary stage		
In situ	0.44 (0.02, 9.20)	0.65 (0.21, 2.05)
Localized	1.00 (Referent)	1.00 (Referent)
Regional/distant	4.39 (0.78, 24.83)	1.98 (0.92, 4.27)
Tumor size (cm)^a^		
<1.0	1.00 (Referent)	1.00 (Referent)
1.0-2.0	2.13 (0.83, 5.44)	*2.92 (1.84, 4.64)*
>2.0	3.17 (0.88, 11.42)	*6.41 (3.30, 12.46)*
Lymph node status^a^		
Negative	1.00 (Referent)	1.00 (Referent)
Positive	1.54 (0.54, 4.34)	1.62 (0.93, 2.81)
Lymphovascular invasion present^a^		
No	1.00 (Referent)	1.00 (Referent)
Yes	1.18 (0.48, 2.87)	1.35 (0.84, 2.18)
Estrogen receptor (ER) status^a^		
ER+	1.00 (Referent)	1.00 (Referent)
ER-	-	1.13 (0.42, 3.07)
Molecular subtype^a,b^		
ER+/PR+/HER2-	1.00 (Referent)	1.00 (Referent)
ER+/PR+/HER2+	1.44 (0.53, 3.91)	1.08 (0.61, 1.91)
ER-/PR-/HER2+	-	1.44 (0.45, 4.63)
ER-/PR-/HER2-	-	1.69 (0.59, 4.82)

Note: odds ratios (ORs) and 95% confidence intervals (Cis) were generated using multivariable models, mutually adjusting for all variables shown in the table as well as for age.

^a^As shown in Table 3, percent unknown for tumor characteristics in the overall study sample was as follows: tumor grade, 17.5%; SEER summary stage, 3.6%; tumor size (cm), 0.1%; lymph node status, 9.8%; lymphovascular invasion present, 19.6%; ER status, 0.3%; molecular subtype, 10.4%.

^b^Molecular subtypes were classified using surrogate classifications, based on immunohistochemical expression of ER and PR, and overexpression or amplification of HER2 (by immunohistochemistry or fluorescence in situ hybridization) as reported in pathology records.

## Data Availability

The sociodemographic, reproductive, clinical, mammographic, and tumor characteristics data used to support the findings of this study may be released upon application to and approval from the Women's Circle of Health Study (WCHS) investigative team, who can be contacted via email at wchstudy@cinj.rutgers.edu. WCHS investigators will make data collected through the study publicly available to outside investigators in compliance with National Institutes of Health (NIH) data sharing policies.

## Supplementary Materials

Supplementary Materials Supplementary Table 1: sensitivity analysis among older women: multivariable logistic regression analysis of the factors associated with mode of detection among Black women aged 50-75 years. Supplementary Table 2: multivariable logistic regression analysis of the factors associated with mode of detection among Black women aged 40-49 years. Supplementary Table 3: multivariable logistic regression analysis of the factors associated with mode of detection among Black women aged 50-59 years. Supplementary Table 4: multivariable logistic regression analysis of the factors associated with mode of detection among Black women aged 60-75 years.
