# Supplementary material for: Factors Associated with Initial Mode of Breast Cancer Detection among Black Women in the Women's Circle of Health Study
Source: J Oncol. 2019 Jul 4;2019:3529651. doi: 10.1155/2019/3529651 (PMC6637674; doi:10.1155/2019/3529651)
Supplement: Supplementary Materials — Supplementary Table 1: sensitivity analysis among older women: multivariable logistic regression analysis of the factors associated with mode of detection among Black women aged 50-75 years. Supplementary Table 2: multivariable logistic regression analysis of the factors associated with mode of detection among Black women aged 40-49 years. Supplementary Table 3: multivariable logistic regression analysis of the factors associated with mode of detection among Black women aged 50-59 years. Supplementary Table 4: multivariable logistic regression analysis of the factors associated with mode of detection among Black women aged 60-75 years. [file 3529651.f1.doc]

| **Supplementary Table 1.** *Sensitivity analysis among older women*: multivariable logistic regression analysis of the factors associated with mode of detection among Black women age 50-75 years | | | |
| --- | --- | --- | --- |
| **Characteristic** | **Clinical breast exam compared to screening mammogram** | **Self-detection compared to screening mammogram** | |
|  | **Multivariable-adjusted** | **Multivariable-adjusted** | |
|  | **OR (95% CI)** | **OR (95% CI)** | |
| Primary health insurance at diagnosis |  |  | |
| Private | 1.00 (Referent) | 1.00 (Referent) | |
| Medicaid | 1.56 (0.35, 7.08) | 1.12 (0.56, 2.26) | |
| Medicare | 2.42 (0.82, 7.10) | 0.64 (0.38, 1.10) | |
| Uninsured | 1.00 (0.17, 6.06) | 1.26 (0.63, 2.52) | |
| Other | - | 0.59 (0.24, 1.49) | |
| Body mass index (kg/m^2^) |  |  | |
| <25.0 | 1.97 (0.52, 7.42) | **2.03 (1.14, 3.62)** | |
| 25.0-29.99 | 1.13 (0.34, 3.74) | 0.80 (0.48, 1.34) | |
| 30.0-34.99 | 1.66 (0.51, 5.44) | 0.67 (0.39, 1.15) | |
| ≥35.0 | 1.00 (Referent) | 1.00 (Referent) | |
| History of hormone therapy use |  |  | |
| No | 1.00 (Referent) | 1.00 (Referent) | |
| Yes | 0.60 (0.21, 1.70) | 0.62 (0.39, 1.00) | |
| Ever had a routine screening mammogram before breast cancer diagnosis |  |  | |
| No | 1.00 (Referent) | 1.00 (Referent) | |
| Yes | **0.12 (0.02, 0.57)** | **0.32 (0.12, 0.83)** | |
| Ever had a doctor perform a clinical breast exam(s) (CBE) before breast cancer diagnosis |  |  | |
| No | 1.00 (Referent) | 1.00 (Referent) | |
| Yes – last CBE performed within the last year | - | **0.45 (0.25, 0.81)** | |
| Yes – last CBE performed more than one year ago | - | 1.06 (0.57, 1.97) | |
| Ever performed breast self-exams (BSEs) before breast cancer diagnosis |  |  | |
| No | 1.00 (Referent) | 1.00 (Referent) | |
| Yes – BSEs performed less than once per month | 1.73 (0.61, 4.89) | **6.66 (3.45, 12.87)** | |
| Yes – BSEs performed at least once per month | **0.26 (0.08, 0.90)** | **6.22 (3.37, 11.49)** | |
| ***Breast tumor clinicopathologic features*** |  |  | |
| Tumor grade^a^ |  |  | |
| Well/moderately differentiated | 1.00 (Referent) | 1.00 (Referent) | |
| Poorly differentiated | 0.42 (0.13, 1.38) | 0.93 (0.57, 1.49) | |
| SEER summary stage |  |  | |
| In situ | -- | 0.53 (0.14, 1.99) | |
| Localized | 1.00 (Referent) | 1.00 (Referent) | |
| Regional/distant | **14.28 (1.26, 162.06)** | 2.08 (0.81, 5.32) | |
| Tumor size (cm)^a^ |  |  | |
| <1.0 | 1.00 (Referent) | 1.00 (Referent) | |
| 1.0-2.0 | 1.33 (0.41, 4.27) | **3.74 (2.08, 6.70)** | |
| >2.0 | 3.15 (0.66, 15.09) | **7.16 (3.21, 15.96)** | |
| Lymph node status^a^ |  |  | |
| Negative | 1.00 (Referent) | 1.00 (Referent) | |
| Positive | 2.38 (0.59, 9.63) | 1.27 (0.66, 2.46) | |
| Lymphovascular invasion present^a^ |  |  | |
| No | 1.00 (Referent) | 1.00 (Referent) | |
| Yes | 1.74 (0.54, 5.58) | 1.65 (0.92, 2.96) | |
| Estrogen receptor (ER) status^a^ |  |  | |
| ER+ | 1.00 (Referent) | 1.00 (Referent) | |
| ER- | - | 0.91 (0.26, 3.17) | |
| Molecular subtype^a,b^ |  |  | |
| ER+/PR+/HER2- | 1.00 (Referent) | 1.00 (Referent) | |
| ER+/PR+/HER2+ |  | 1.03 (0.52, 2.07) | |
| ER-/PR-/HER2+ | - | 1.78 (0.41, 7.68) | |
| ER-/PR-/HER2- | - | 2.13 (0.59, 7.70) | |
| NOTE: Odds ratios (ORs) and 95% confidence intervals (Cis) were generated using multivariable models, mutually adjusting for all variables shown in the table as well as for age.  ^a^ As shown in Table 3, percent unknown for tumor characteristics in the overall study sample were the following: tumor grade, 17.5%; SEER summary stage, 3.6%; tumor size (cm), 0.1%; lymph node status, 9.8%; lymphovascular invasion present, 19.6%; ER status, 0.3%; molecular subtype, 10.4%. | | |  |
| ^b^ Molecular subtypes were classified using surrogate classifications, based on immunohistochemical expression of ER and PR, and overexpression or amplification of HER2 (by immunohistochemistry or fluorescence in situ hybridization) as reported in pathology records. | | |  |

| **Supplementary Table 2.** Multivariable logistic regression analysis of the factors associated with mode of detection among Black women age 40-49 years | | | |
| --- | --- | --- | --- |
| **Characteristic** | **Clinical breast exam compared to screening mammogram** | **Self-detection compared to screening mammogram** | |
|  | **Multivariable-adjusted** | **Multivariable-adjusted** | |
|  | **OR (95% CI)** | **OR (95% CI)** | |
| Primary health insurance at diagnosis |  |  | |
| Private | 1.00 (Referent) | 1.00 (Referent) | |
| Medicaid | 0.47 (0.06, 3.51) | 0.81 (0.27, 2.37) | |
| Medicare | - | 1.15 (0.06, 22.86) | |
| Uninsured | 0.16 (0.01, 3.13) | 2.16 (0.56, 8.33) | |
| Other | 0.50 (0.03, 9.54) | 1.19 (0.32, 4.39) | |
| Body mass index (kg/m^2^) |  |  | |
| <25.0 | 1.86 (0.29, 11.94) | **8.02 (2.77, 23.21)** | |
| 25.0-29.99 | 0.60 (0.12, 3.12) | **3.12 (1.24, 7.88)** | |
| 30.0-34.99 | 1.43 (0.23, 8.86) | 1.80 (0.61, 5.26) | |
| ≥35.0 | 1.00 (Referent) | 1.00 (Referent) | |
| History of hormone therapy use |  |  | |
| No | 1.00 (Referent) | 1.00 (Referent) | |
| Yes | - | 0.19 (0.03, 1.18) | |
| Ever had a routine screening mammogram before breast cancer diagnosis |  |  | |
| No | 1.00 (Referent) | 1.00 (Referent) | |
| Yes | 0.21 (0.04, 1.14) | 0.56 (0.18, 1.72) | |
| Ever had a doctor perform a clinical breast exam(s) (CBE) before breast cancer diagnosis |  |  | |
| No | 1.00 (Referent) | 1.00 (Referent) | |
| Yes – last CBE performed within the last year | 0.81 (0.1, 6.31) | 0.64 (0.2, 2.02) | |
| Yes – last CBE performed more than one year ago | 5.17 (0.61, 44.06) | 2.74 (0.73, 10.36) | |
| Ever performed breast self-exams (BSEs) before breast cancer diagnosis |  |  | |
| No | 1.00 (Referent) | 1.00 (Referent) | |
| Yes – BSEs performed less than once per month | 0.21 (0.04, 1.16) | 1.44 (0.55, 3.78) | |
| Yes – BSEs performed at least once per month | 0.42 (0.09, 1.92) | **4.88 (2.00, 11.89)** | |
| ***Breast tumor clinicopathologic features*** |  |  | |
| Tumor grade^a^ |  |  | |
| Well/moderately differentiated | 1.00 (Referent) | 1.00 (Referent) | |
| Poorly differentiated | 1.05 (0.24, 4.53) | 2.04 (0.9, 4.65) | |
| SEER summary stage |  |  | |
| In situ | 0.22 (0.00, 58.67) | 1.01 (0.08, 13.44) | |
| Localized | 1.00 (Referent) | 1.00 (Referent) | |
| Regional/distant | 3.32 (0.11, 102.23) | 2.02 (0.36, 11.20) | |
| Tumor size (cm)^a^ |  |  | |
| <1.0 | 1.00 (Referent) | 1.00 (Referent) | |
| 1.0-2.0 | **21.87 (1.46, 328.47)** | 1.91 (0.78, 4.68) | |
| >2.0 | 5.39 (0.16, 182.31) | **10.57 (2.12, 52.71)** | |
| Lymph node status^a^ |  |  | |
| Negative | 1.00 (Referent) | 1.00 (Referent) | |
| Positive | 1.23 (0.09, 17.53) | **5.41 (1.45, 20.15)** | |
| Lymphovascular invasion present^a^ |  |  | |
| No | 1.00 (Referent) | 1.00 (Referent) | |
| Yes | 0.43 (0.07, 2.76) | 0.74 (0.28, 1.96) | |
| Estrogen receptor (ER) status^a^ |  |  | |
| ER+ | 1.00 (Referent) | 1.00 (Referent) | |
| ER- | - | 2.49 (0.27, 22.92) | |
| Molecular subtype^a,b^ |  |  | |
| ER+/PR+/HER2- | 1.00 (Referent) | 1.00 (Referent) | |
| ER+/PR+/HER2+ | **9.27 (1.49, 57.82)** | 1.74 (0.51, 5.99) | |
| ER-/PR-/HER2+ | - | 0.76 (0.06, 9.33) | |
| ER-/PR-/HER2- | - | 1.02 (0.10, 10.08) | |
| NOTE: Odds ratios (ORs) and 95% confidence intervals (Cis) were generated using multivariable models, mutually adjusting for all variables shown in the table as well as age.  ^a^ As shown in Table 3, percent unknown for tumor characteristics in the overall study sample were the following: tumor grade, 17.5%; SEER summary stage, 3.6%; tumor size (cm), 0.1%; lymph node status, 9.8%; lymphovascular invasion present, 19.6%; ER status, 0.3%; molecular subtype, 10.4%. | | |  |
| ^b^ Molecular subtypes were classified using surrogate classifications, based on immunohistochemical expression of ER and PR, and overexpression or amplification of HER2 (by immunohistochemistry or fluorescence in situ hybridization) as reported in pathology records. | | |  |

| **Supplementary Table 3.** Multivariable logistic regression analysis of the factors associated with mode of detection among Black women age 50-59 years | | | |
| --- | --- | --- | --- |
| **Characteristic** | **Clinical breast exam compared to screening mammogram** | **Self-detection compared to screening mammogram** | |
|  | **Multivariable-adjusted** | **Multivariable-adjusted** | |
|  | **OR (95% CI)** | **OR (95% CI)** | |
| Primary health insurance at diagnosis |  |  | |
| Private | 1.00 (Referent) | 1.00 (Referent) | |
| Medicaid | 1.14 (0.10, 13.63) | 1.21 (0.42, 3.50) | |
| Medicare | 24.32 (0.96, 617.58) | 0.60 (0.11, 3.4) | |
| Uninsured | - | 1.95 (0.71, 5.41) | |
| Other | - | 0.59 (0.17, 2.02) | |
| Body mass index (kg/m^2^) |  |  | |
| <25.0 | **22.45 (1.25, 401.86)** | **2.53 (1.06, 6.01)** | |
| 25.0-29.99 | **21.78 (1.12, 423.05)** | 1.30 (0.60, 2.85) | |
| 30.0-34.99 | 17.88 (0.94, 339.22) | 0.65 (0.28, 1.53) | |
| ≥35.0 | 1.00 (Referent) | 1.00 (Referent) | |
| History of hormone therapy use |  |  | |
| No | 1.00 (Referent) | 1.00 (Referent) | |
| Yes | 0.20 (0.02, 2.11) | 0.52 (0.23, 1.18) | |
| Ever had a routine screening mammogram before breast cancer diagnosis |  |  | |
| No | 1.00 (Referent) | 1.00 (Referent) | |
| Yes | **0.05 (0.00, 0.70)** | 0.33 (0.08, 1.29) | |
| Ever had a doctor perform a clinical breast exam(s) (CBE) before breast cancer diagnosis |  |  | |
| No | 1.00 (Referent) | 1.00 (Referent) | |
| Yes – last CBE performed within the last year | - | **0.30 (0.10, 0.86)** | |
| Yes – last CBE performed more than one year ago | - | 0.71 (0.23, 2.16) | |
| Ever performed breast self-exams (BSEs) before breast cancer diagnosis |  |  | |
| No | 1.00 (Referent) | 1.00 (Referent) | |
| Yes – BSEs performed less than once per month | **7.29 (1.16, 45.64)** | **17.4 (5.71, 52.99)** | |
| Yes – BSEs performed at least once per month | 0.19 (0.01, 2.74) | **14.42 (5.00, 41.61)** | |
| ***Breast tumor clinicopathologic features*** |  |  | |
| Tumor grade^a^ |  |  | |
| Well/moderately differentiated | 1.00 (Referent) | 1.00 (Referent) | |
| Poorly differentiated | 1.36 (0.16, 11.6) | 1.26 (0.62, 2.57) | |
| SEER summary stage |  |  | |
| In situ | -- | 0.68 (0.07, 6.80) | |
| Localized | 1.00 (Referent) | 1.00 (Referent) | |
| Regional/distant | 1.09 (0.00, 789.68) | 1.61 (0.33, 7.90) | |
| Tumor size (cm)^a^ |  |  | |
| <1.0 | 1.00 (Referent) | 1.00 (Referent) | |
| 1.0-2.0 | 0.34 (0.05, 2.29) | **2.93 (1.21, 7.14)** | |
| >2.0 | 2.72 (0.19, 38.24) | **9.47 (2.67, 33.59)** | |
| Lymph node status^a^ |  |  | |
| Negative | 1.00 (Referent) | 1.00 (Referent) | |
| Positive | 17.25 (0.99, 299.31) | 2.24 (0.78, 6.44) | |
| Lymphovascular invasion present^a^ |  |  | |
| No | 1.00 (Referent) | 1.00 (Referent) | |
| Yes | 1.75 (0.28, 11.00) | 1.52 (0.66, 3.53) | |
| Estrogen receptor (ER) status^a^ |  |  | |
| ER+ | 1.00 (Referent) | 1.00 (Referent) | |
| ER- | - | 1.79 (0.35, 9.04) | |
| Molecular subtype^a,b^ |  |  | |
| ER+/PR+/HER2- | 1.00 (Referent) | 1.00 (Referent) | |
| ER+/PR+/HER2+ | 0.48 (0.04, 6.74) | 0.94 (0.36, 2.46) | |
| ER-/PR-/HER2+ | - | 0.91 (0.12, 7.02) | |
| ER-/PR-/HER2- | - | 1.39 (0.24, 8.00) | |
| NOTE: Odds ratios (ORs) and 95% confidence intervals (Cis) were generated using multivariable models, mutually adjusting for all variables shown in the table as well as age.  ^a^ As shown in Table 3, percent unknown for tumor characteristics in the overall study sample were the following: tumor grade, 17.5%; SEER summary stage, 3.6%; tumor size (cm), 0.1%; lymph node status, 9.8%; lymphovascular invasion present, 19.6%; ER status, 0.3%; molecular subtype, 10.4%. | | |  |
| ^b^ Molecular subtypes were classified using surrogate classifications, based on immunohistochemical expression of ER and PR, and overexpression or amplification of HER2 (by immunohistochemistry or fluorescence in situ hybridization) as reported in pathology records. | | |  |

| **Supplementary Table 4.** Multivariable logistic regression analysis of the factors associated with mode of detection among Black women age 60-75 years | | | |
| --- | --- | --- | --- |
| **Characteristic** | **Clinical breast exam compared to screening mammogram** | **Self-detection compared to screening mammogram** | |
|  | **Multivariable-adjusted** | **Multivariable-adjusted** | |
|  | **OR (95% CI)** | **OR (95% CI)** | |
| Primary health insurance at diagnosis |  |  | |
| Private | 1.00 (Referent) | 1.00 (Referent) | |
| Medicaid | 2.53 (0.23, 28.19) | 0.80 (0.28, 2.31) | |
| Medicare | 2.69 (0.49, 14.78) | 0.65 (0.34, 1.26) | |
| Uninsured | 5.1 (0.5, 52.03) | 0.81 (0.28, 2.34) | |
| Other | - | 0.67 (0.16, 2.86) | |
| Body mass index (kg/m^2^) |  |  | |
| <25.0 | 0.59 (0.05, 7.17) | 2.03 (0.86, 4.78) | |
| 25.0-29.99 | 0.18 (0.02, 2.02) | 0.52 (0.24, 1.13) | |
| 30.0-34.99 | 0.69 (0.13, 3.66) | 0.75 (0.36, 1.58) | |
| ≥35.0 | 1.00 (Referent) | 1.00 (Referent) | |
| History of hormone therapy use |  |  | |
| No | 1.00 (Referent) | 1.00 (Referent) | |
| Yes |  |  | |
| Ever had a routine screening mammogram before breast cancer diagnosis |  |  | |
| No | 1.00 (Referent) | 1.00 (Referent) | |
| Yes | 0.24 (0.01, 12.15) | 0.27 (0.06, 1.23) | |
| Ever had a doctor perform a clinical breast exam(s) (CBE) before breast cancer diagnosis |  |  | |
| No | 1.00 (Referent) | 1.00 (Referent) | |
| Yes – last CBE performed within the last year | - | 0.47 (0.21, 1.04) | |
| Yes – last CBE performed more than one year ago | - | 1.34 (0.60, 3.01) | |
| Ever performed breast self-exams (BSEs) before breast cancer diagnosis |  |  | |
| No | 1.00 (Referent) | 1.00 (Referent) | |
| Yes – BSEs performed less than once per month | 0.29 (0.04, 2.43) | **3.80 (1.55, 9.27)** | |
| Yes – BSEs performed at least once per month | 0.19 (0.03, 1.18) | **3.71 (1.62, 8.46)** | |
| ***Breast tumor clinicopathologic features*** |  |  | |
| Tumor grade^a^ |  |  | |
| Well/moderately differentiated | 1.00 (Referent) | 1.00 (Referent) | |
| Poorly differentiated | 0.28 (0.03, 2.27) | 0.66 (0.33, 1.35) | |
| SEER summary stage |  |  | |
| In situ | -- | 0.60 (0.10, 3.50) | |
| Localized | 1.00 (Referent) | 1.00 (Referent) | |
| Regional/distant | 5.14 (0.16, 168.79) | 1.68 (0.45, 6.25) | |
| Tumor size (cm)^a^ |  |  | |
| <1.0 | 1.00 (Referent) | 1.00 (Referent) | |
| 1.0-2.0 | 4.07 (0.39, 42.96) | **4.71 (1.98, 11.2)** | |
| >2.0 | 25.84 (0.81, 824.36) | **10.12 (2.92, 35.07)** | |
| Lymph node status^a^ |  |  | |
| Negative | 1.00 (Referent) | 1.00 (Referent) | |
| Positive | 1.44 (0.15, 14.11) | 0.87 (0.34, 2.25) | |
| Lymphovascular invasion present^a^ |  |  | |
| No | 1.00 (Referent) | 1.00 (Referent) | |
| Yes | 3.53 (0.47, 26.51) | 2.09 (0.87, 5.05) | |
| Estrogen receptor (ER) status^a^ |  |  | |
| ER+ | 1.00 (Referent) | 1.00 (Referent) | |
| ER- | - | 0.4 (0.05, 3.32) | |
| Molecular subtype^a,b^ |  |  | |
| ER+/PR+/HER2- | 1.00 (Referent) | 1.00 (Referent) | |
| ER+/PR+/HER2+ | 2.16 (0.16, 29.64) | 1.18 (0.37, 3.76) | |
| ER-/PR-/HER2+ | - | 2.74 (0.22, 33.37) | |
| ER-/PR-/HER2- | - | 3.67 (0.43, 31.11) | |
| NOTE: Odds ratios (ORs) and 95% confidence intervals (Cis) were generated using multivariable models, mutually adjusting for all variables shown in the table as well as age.  ^a^ As shown in Table 3, percent unknown for tumor characteristics in the overall study sample were the following: tumor grade, 17.5%; SEER summary stage, 3.6%; tumor size (cm), 0.1%; lymph node status, 9.8%; lymphovascular invasion present, 19.6%; ER status, 0.3%; molecular subtype, 10.4%. | | |  |
| ^b^ Molecular subtypes were classified using surrogate classifications, based on immunohistochemical expression of ER and PR, and overexpression or amplification of HER2 (by immunohistochemistry or fluorescence in situ hybridization) as reported in pathology records. | | |  |
